# *N*,*N*′-Bis(4-bromo­phen­yl)pyridine-2,6-dicarboxamide

**DOI:** 10.1107/S1600536813002705

**Published:** 2013-02-20

**Authors:** Ghulam Waris, Humaira Masood Siddiqi, Ulrich Flörke, Rizwan Hussain, M. Saeed Butt

**Affiliations:** a Department of Chemistry, Quaid-I-Azam University, Islamabad 45320, Pakistan; b Universität Paderborn, Warburgerstrasse 100, D-33098 Paderborn, Germany; c NESCOM, PO Box 2216 Islamabad, Pakistan

## Abstract

The mol­ecule of the title compound, C_19_H_13_Br_2_N_3_O_2_, lies about a twofold rotation axis. The benzene ring makes dihedral angles of 8.9 (2) and 16.4 (2)° with the central pyridine ring and the second benzene ring, respectively. An intra­molecular N—H⋯N contact occurs. In the crystal, mol­ecules are connected by pairs of N—H⋯O hydrogen bonds into chains along the *c* axis.

## Related literature

For related structures, see: Malone *et al.* (1997 ▶); Qi *et al.* (2003 ▶). For imide–amide polymers, see: Sun *et al.* (2006 ▶); Zhong *et al.* (2002 ▶). For properties of polymers containing heterocyclic groups, see: Diakoumakos & Mikroyannidis (1994 ▶); Hamciuc *et al.* (2001 ▶).
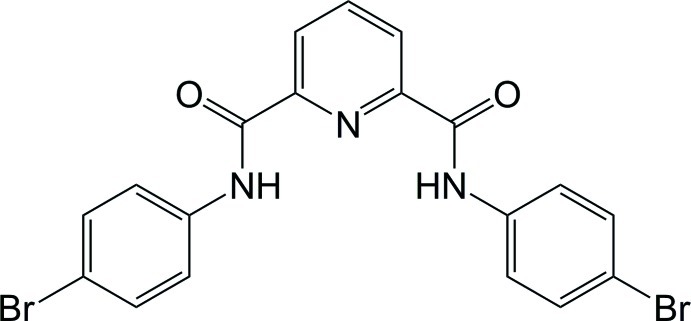


## Experimental

### 

#### Crystal data

C_19_H_13_Br_2_N_3_O_2_
*M* *_r_* = 475.14
Monoclinic, 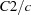

*a* = 9.550 (2) Å
*b* = 22.698 (5) Å
*c* = 8.748 (2) Å
β = 107.511 (5)°
*V* = 1808.5 (7) Å^3^
*Z* = 4
Mo *K*α radiation
μ = 4.50 mm^−1^
*T* = 130 K
0.21 × 0.12 × 0.11 mm

#### Data collection

Bruker SMART APEX diffractometer
Absorption correction: multi-scan (*SADABS*; Bruker, 2002 ▶) *T*_min_ = 0.452, *T*_max_ = 0.637
8520 measured reflections
2159 independent reflections
1695 reflections with *I* > 2σ(*I*)
*R*_int_ = 0.044

#### Refinement

*R*[*F* ^2^ > 2σ(*F* ^2^)] = 0.039
*wR*(*F* ^2^) = 0.105
*S* = 1.02
2159 reflections
119 parameters
H-atom parameters constrained
Δρ_max_ = 0.94 e Å^−3^
Δρ_min_ = −0.52 e Å^−3^

### 

Data collection: *SMART* (Bruker, 2002 ▶); cell refinement: *SAINT* (Bruker, 2002 ▶); data reduction: *SAINT*; program(s) used to solve structure: *SHELXTL* (Sheldrick, 2008 ▶); program(s) used to refine structure: *SHELXTL*; molecular graphics: *SHELXTL*; software used to prepare material for publication: *SHELXTL* and local programs.

## Supplementary Material

Crystal structure: contains datablock(s) I, global. DOI: 10.1107/S1600536813002705/yk2085sup1.cif

Structure factors: contains datablock(s) I. DOI: 10.1107/S1600536813002705/yk2085Isup2.hkl

Supplementary material file. DOI: 10.1107/S1600536813002705/yk2085Isup3.cml

Additional supplementary materials:  crystallographic information; 3D view; checkCIF report

## Figures and Tables

**Table 1:** Hydrogen-bond geometry (Å, °)

*D*—H⋯*A*	*D*—H	H⋯*A*	*D*⋯*A*	*D*—H⋯*A*
N1—H1*A*⋯N2	0.88	2.23	2.673 (3)	111
N1—H1*A*⋯O1^i^	0.88	2.32	3.044 (3)	140

Symmetry code: (i) 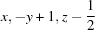.

**Fig. 1.**
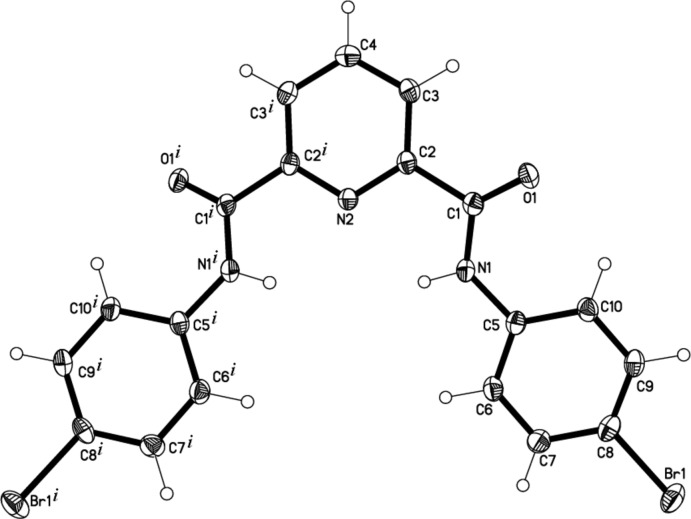
Molecular structure of the title compound with anisotropic displacement ellipsoids drawn at the 50% probability level. Symmetry code: (i) -x+1, y, -z+3/2.

**Fig. 2.**
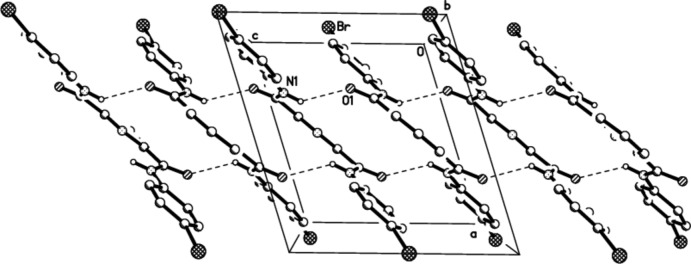
Crystal packing viewd along b axis with hydrogen bonds as dotted lines. H-atoms not involved are omitted.

## supplementary crystallographic information

### Comment

Aromatic Poly(amide-imide)s are classified as *meta* aramid family. They are non-flammable, which is a permanent characteristic of their chemical structure. It includes a high proportion of aromatic groups and combined double bonds. The demand for polyamide-imide (PAI) and other high-temperature resistant polymeric materials has grown steadily because of their outstanding mechanical properties, excellent thermal and oxidative stability (Zhong *et al.*, 2002; Sun *et al.*, 2006). Incorporation of heterocylic groups in the polymer backbone is a rational approach which promotes solubility without affecting thermal and mechanical properties to any great extent (Diakoumakos *et al.*, 1994, Hamciuc *et al.*, 2001). As part of our enduring interest in solubility of aromatic poly(amide-imide)s by structural modification, we are reporting a pyridine-based monomer having inbuilt amide functionality. It enhances the solubility of resulting poly(amid-imide)s without worsening the inherent properties of the polymer.

### Experimental

In this preparation, chemicals of reagent grade quality were used without their further purification. In a 100 ml three-necked round-bottomed flask, equipped with a condenser, a nitrogen gas inlet tube, a thermometer and a magnetic stirrer, 0.02 mole (3.44 g) of 4-bromoaniline in 25 mL of dry tetrahydrofuran (THF) were stirred at 273–278 K for 30 minutes and 0.01 mol (2.04 g) of pyridine-2,6-dicarbonyl dichloride in 30 mL of THF was added dropwise by dropping funnel. Stirring was continued for further 1 h at the same conditions. The temperature of reaction mixture was then raised to 308–313 K and stirring was continued for 45 minutes. The flask content was cooled to room temperature, poured into water and left for 24 h. Resulting dark brown precipitate was filtered, washed with hot water and 5% NaOH solution. Finally, product was washed with hot water and methanol, dried under vacuum at 353 K. The crude product was recrystallized from THF-ethylacetate mixture (1:2).

### Refinement

Hydrogen atoms were identified in difference syntheses, and then refined at idealized positions riding on the carbon or nitrogen atoms with C—H = 0.95 Å and N—H = 0.88 Å and isotropic displacement parameters *U*_iso_(H) = 1.2U(C/N_eq_).

### Crystal data

**Table:**

C_19_H_13_Br_2_N_3_O_2_	*F*(000) = 936
*M**_r_* = 475.14	*D*_x_ = 1.745 Mg m^−^^3^
Monoclinic, *C*2/*c*	Mo *K*α radiation, λ = 0.71073 Å
Hall symbol: -C 2yc	Cell parameters from 1650 reflections
*a* = 9.550 (2) Å	θ = 2.9–23.6°
*b* = 22.698 (5) Å	µ = 4.50 mm^−^^1^
*c* = 8.748 (2) Å	*T* = 130 K
β = 107.511 (5)°	Prism, colourless
*V* = 1808.5 (7) Å^3^	0.21 × 0.12 × 0.11 mm
*Z* = 4	

### Data collection

**Table:**

Bruker SMART APEX diffractometer	2159 independent reflections
Radiation source: sealed tube	1695 reflections with *I* > 2σ(*I*)
Graphite monochromator	*R*_int_ = 0.044
φ and ω scans	θ_max_ = 27.9°, θ_min_ = 1.8°
Absorption correction: multi-scan (*SADABS*; Bruker, 2002)	*h* = −12→12
*T*_min_ = 0.452, *T*_max_ = 0.637	*k* = −29→29
8520 measured reflections	*l* = −11→11

### Refinement

**Table:**

Refinement on *F*^2^	Primary atom site location: structure-invariant direct methods
Least-squares matrix: full	Secondary atom site location: difference Fourier map
*R*[*F*^2^ > 2σ(*F*^2^)] = 0.039	Hydrogen site location: difference Fourier map
*wR*(*F*^2^) = 0.105	H-atom parameters constrained
*S* = 1.02	*w* = 1/[σ^2^(*F*_o_^2^) + (0.0601*P*)^2^ + 0.9564*P*] where *P* = (*F*_o_^2^ + 2*F*_c_^2^)/3
2159 reflections	(Δ/σ)_max_ < 0.001
119 parameters	Δρ_max_ = 0.94 e Å^−^^3^
0 restraints	Δρ_min_ = −0.52 e Å^−^^3^

### Special details

**Table:**

Geometry. All s.u.'s (except the s.u. in the dihedral angle between two l.s. planes) are estimated using the full covariance matrix. The cell s.u.'s are taken into account individually in the estimation of s.u.'s in distances, angles and torsion angles; correlations between s.u.'s in cell parameters are only used when they are defined by crystal symmetry. An approximate (isotropic) treatment of cell s.u.'s is used for estimating s.u.'s involving l.s. planes.
Refinement. Refinement of *F*^2^ against ALL reflections. The weighted *R*-factor *wR* and goodness of fit *S* are based on *F*^2^, conventional *R*-factors *R* are based on *F*, with *F* set to zero for negative *F*^2^. The threshold expression of *F*^2^ > σ(*F*^2^) is used only for calculating *R*-factors(gt) *etc*. and is not relevant to the choice of reflections for refinement. *R*-factors based on *F*^2^ are statistically about twice as large as those based on *F*, and *R*- factors based on ALL data will be even larger.

### Fractional atomic coordinates and isotropic or equivalent isotropic displacement parameters (Å^2^)

**Table:**

	*x*	*y*	*z*	*U*_iso_*/*U*_eq_	
Br1	−0.04692 (4)	0.721218 (15)	1.02697 (4)	0.03818 (15)	
O1	0.2856 (2)	0.44490 (9)	1.0115 (3)	0.0268 (5)	
N1	0.3088 (3)	0.52384 (11)	0.8566 (3)	0.0225 (5)	
H1A	0.3497	0.5339	0.7828	0.027*	
N2	0.5000	0.46292 (15)	0.7500	0.0199 (7)	
C1	0.3308 (3)	0.46760 (13)	0.9081 (4)	0.0214 (6)	
C2	0.4206 (3)	0.43212 (13)	0.8256 (4)	0.0210 (6)	
C3	0.4179 (3)	0.37121 (13)	0.8303 (4)	0.0243 (6)	
H3A	0.3615	0.3510	0.8867	0.029*	
C4	0.5000	0.34048 (19)	0.7500	0.0273 (9)	
H4A	0.5000	0.2986	0.7500	0.033*	
C5	0.2290 (3)	0.56882 (13)	0.9052 (4)	0.0218 (6)	
C6	0.2466 (3)	0.62624 (14)	0.8597 (4)	0.0259 (7)	
H6A	0.3134	0.6342	0.8008	0.031*	
C7	0.1690 (4)	0.67177 (14)	0.8988 (4)	0.0278 (7)	
H7A	0.1827	0.7110	0.8685	0.033*	
C8	0.0705 (3)	0.65961 (15)	0.9830 (4)	0.0261 (7)	
C9	0.0511 (3)	0.60299 (15)	1.0301 (4)	0.0269 (7)	
H9A	−0.0168	0.5954	1.0879	0.032*	
C10	0.1312 (3)	0.55712 (14)	0.9927 (4)	0.0252 (7)	
H10A	0.1198	0.5181	1.0263	0.030*	

### Atomic displacement parameters (Å^2^)

**Table:**

	*U*^11^	*U*^22^	*U*^33^	*U*^12^	*U*^13^	*U*^23^
Br1	0.0469 (3)	0.0388 (2)	0.0340 (2)	0.01775 (15)	0.01988 (18)	0.00280 (15)
O1	0.0261 (12)	0.0298 (12)	0.0281 (12)	−0.0035 (9)	0.0136 (10)	0.0022 (9)
N1	0.0198 (13)	0.0268 (13)	0.0233 (13)	0.0011 (10)	0.0100 (11)	0.0006 (10)
N2	0.0142 (17)	0.0228 (17)	0.0212 (18)	0.000	0.0029 (14)	0.000
C1	0.0129 (14)	0.0262 (15)	0.0232 (15)	−0.0029 (11)	0.0024 (11)	−0.0021 (12)
C2	0.0136 (14)	0.0262 (15)	0.0220 (15)	−0.0010 (11)	0.0037 (12)	0.0003 (12)
C3	0.0201 (15)	0.0281 (15)	0.0246 (15)	−0.0032 (12)	0.0068 (12)	0.0011 (13)
C4	0.028 (2)	0.022 (2)	0.029 (2)	0.000	0.0058 (19)	0.000
C5	0.0159 (14)	0.0280 (15)	0.0206 (15)	0.0010 (11)	0.0041 (12)	−0.0008 (12)
C6	0.0253 (16)	0.0305 (16)	0.0245 (16)	0.0020 (13)	0.0115 (13)	0.0039 (13)
C7	0.0332 (18)	0.0262 (15)	0.0250 (16)	0.0058 (13)	0.0100 (14)	0.0054 (13)
C8	0.0198 (15)	0.0341 (17)	0.0219 (16)	0.0094 (12)	0.0027 (12)	−0.0007 (12)
C9	0.0186 (15)	0.0367 (17)	0.0267 (16)	0.0026 (12)	0.0087 (13)	0.0006 (14)
C10	0.0192 (15)	0.0283 (15)	0.0291 (17)	−0.0016 (12)	0.0087 (13)	0.0003 (13)

### Geometric parameters (Å, º)

**Table:**

Br1—C8	1.903 (3)	C4—H4A	0.9500
O1—C1	1.227 (4)	C5—C6	1.387 (4)
N1—C1	1.349 (4)	C5—C10	1.400 (4)
N1—C5	1.414 (4)	C6—C7	1.373 (4)
N1—H1A	0.8800	C6—H6A	0.9500
N2—C2^i^	1.343 (3)	C7—C8	1.387 (4)
N2—C2	1.343 (3)	C7—H7A	0.9500
C1—C2	1.509 (4)	C8—C9	1.379 (5)
C2—C3	1.384 (4)	C9—C10	1.388 (4)
C3—C4	1.388 (4)	C9—H9A	0.9500
C3—H3A	0.9500	C10—H10A	0.9500
C4—C3^i^	1.388 (4)		
			
C1—N1—C5	128.9 (3)	C6—C5—N1	118.0 (3)
C1—N1—H1A	115.6	C10—C5—N1	122.4 (3)
C5—N1—H1A	115.6	C7—C6—C5	121.0 (3)
C2^i^—N2—C2	117.3 (3)	C7—C6—H6A	119.5
O1—C1—N1	126.1 (3)	C5—C6—H6A	119.5
O1—C1—C2	120.3 (3)	C6—C7—C8	119.1 (3)
N1—C1—C2	113.5 (3)	C6—C7—H7A	120.5
N2—C2—C3	123.5 (3)	C8—C7—H7A	120.5
N2—C2—C1	116.4 (3)	C9—C8—C7	121.2 (3)
C3—C2—C1	120.1 (3)	C9—C8—Br1	119.0 (2)
C2—C3—C4	118.0 (3)	C7—C8—Br1	119.8 (2)
C2—C3—H3A	121.0	C8—C9—C10	119.7 (3)
C4—C3—H3A	121.0	C8—C9—H9A	120.2
C3—C4—C3^i^	119.7 (4)	C10—C9—H9A	120.2
C3—C4—H4A	120.2	C9—C10—C5	119.5 (3)
C3^i^—C4—H4A	120.2	C9—C10—H10A	120.3
C6—C5—C10	119.6 (3)	C5—C10—H10A	120.3
			
C5—N1—C1—O1	−1.1 (5)	C1—N1—C5—C10	−14.8 (5)
C5—N1—C1—C2	179.0 (3)	C10—C5—C6—C7	−0.3 (5)
C2^i^—N2—C2—C3	0.6 (2)	N1—C5—C6—C7	178.0 (3)
C2^i^—N2—C2—C1	−179.3 (3)	C5—C6—C7—C8	−0.8 (5)
O1—C1—C2—N2	−161.6 (2)	C6—C7—C8—C9	1.0 (5)
N1—C1—C2—N2	18.3 (4)	C6—C7—C8—Br1	−176.2 (2)
O1—C1—C2—C3	18.5 (4)	C7—C8—C9—C10	−0.1 (5)
N1—C1—C2—C3	−161.6 (3)	Br1—C8—C9—C10	177.1 (2)
N2—C2—C3—C4	−1.1 (4)	C8—C9—C10—C5	−1.1 (5)
C1—C2—C3—C4	178.8 (2)	C6—C5—C10—C9	1.3 (5)
C2—C3—C4—C3^i^	0.5 (2)	N1—C5—C10—C9	−177.0 (3)
C1—N1—C5—C6	166.9 (3)		

Symmetry code: (i) −*x*+1, *y*, −*z*+3/2.

### Hydrogen-bond geometry (Å, º)

**Table:**

*D*—H···*A*	*D*—H	H···*A*	*D*···*A*	*D*—H···*A*
N1—H1*A*···N2	0.88	2.23	2.673 (3)	111
N1—H1*A*···O1^ii^	0.88	2.32	3.044 (3)	140

Symmetry code: (ii) *x*, −*y*+1, *z*−1/2.
